# The NPH radscale; a new radiological scale for evaluation of suspected normal pressure hydrocephalus

**DOI:** 10.1186/2045-8118-12-S1-P27

**Published:** 2015-09-18

**Authors:** Karin Kockum, Elna-Marie Larsson, Otto Lilja-Lund, Michelle Rosell, Lars Söderström, Johan Virhammar, Katarina Laurell

**Affiliations:** 1 Department of Pharmacology and Clinical Neuroscience, Unit of Research, Education and Development, Östersund, Umeå University, Sweden; 2 Department of Surgical Sciences, Radiology, Uppsala University, Sweden; 3 Unit of Research, Education and Development, östersund Hospital, Region Jämtland Härjedalen, Sweden; 4 Dept of Neuroscience, Neurology, Uppsala University Hospital, Sweden

## Introduction

Imaging of the brain with computerized tomography (CT) or magnetic resonance imaging (MRI) is crucial to support the diagnosis of normal pressure hydrocephalus (NPH). The aim of this study was to construct a radiological scale, composed of morphological signs of NPH, and compare with an existing scale for clinical NPH symptoms developed by Hellström et al[1].

## Methods

In a prospective population-based study of the prevalence of NPH, 91 individuals (43 males), mean age 74 years (range 66-92 years), underwent CT of the brain and neurological examination with assessment of clinical symptoms. A radiological scale consisting of eight radiological parameters was developed and correlated with the clinical NPH scale score to yield a reliable diagnostic tool. Two independent radiologists, blinded to clinical data, visually assessed and performed all measurements of the parameters Evans index >0.3, callosal angle <90°, narrow high convexity sulci, focally dilated sulci, dilated Sylvian fissures, focal bulging of ventricular roof, dilated temporal horns, and periventricular hyperintensities. After conversion into points, the parameters were summarized yielding a score ranging from 0 to 10 points where higher scores indicates more severe radiological changes.

## Results

Mean clinical NPH score was 83 (min 30, max 100, SD=17). Mean NPH radscale was 2 (min 0, max 10, SD=2). There was a significant correlation (rs=0.5, p<0.0001) between the new NPH radscale and clinical NPH symptoms as assessed by the NPH symptom scale.

## Conclusion

The new NPH radscale seems to be a promising tool for diagnosing NPH.
